# COVID‑19 detection from chest X-ray images using transfer learning

**DOI:** 10.1038/s41598-024-61693-0

**Published:** 2024-05-21

**Authors:** Enas M. F. El Houby

**Affiliations:** Systems and Information Department, National Research Centre, Dokki, 12311 Cairo Egypt

**Keywords:** Classification, Convolution neural network, Coronavirus, COVID-19, Deep learning, Transfer learning, Computational biology and bioinformatics, Diseases, Engineering

## Abstract

COVID-19 is a kind of coronavirus that appeared in China in the Province of Wuhan in December 2019. The most significant influence of this virus is its very highly contagious characteristic which may lead to death. The standard diagnosis of COVID-19 is based on swabs from the throat and nose, their sensitivity is not high enough and so they are prone to errors. Early diagnosis of COVID-19 disease is important to provide the chance of quick isolation of the suspected cases and to decrease the opportunity of infection in healthy people. In this research, a framework for chest X-ray image classification tasks based on deep learning is proposed to help in early diagnosis of COVID-19. The proposed framework contains two phases which are the pre-processing phase and classification phase which uses pre-trained convolution neural network models based on transfer learning. In the pre-processing phase, different image enhancements have been applied to full and segmented X-ray images to improve the classification performance of the CNN models. Two CNN pre-trained models have been used for classification which are VGG19 and EfficientNetB0. From experimental results, the best model achieved a sensitivity of 0.96, specificity of 0.94, precision of 0.9412, F1 score of 0.9505 and accuracy of 0.95 using enhanced full X-ray images for binary classification of chest X-ray images into COVID-19 or normal with VGG19. The proposed framework is promising and achieved a classification accuracy of 0.935 for 4-class classification.

## Introduction

Since December 2019, coronavirus has been disseminated from China to many other countries. Coronavirus which is called SARS-CoV-2 causes COVID-19 as named by World Health Organization (WHO) on February 11, 2020. World Health Organization announced COVID-19 disease resulted from the coronavirus as a world pandemic in March 2020^1^. The disease has disseminated to nearly all countries, resulting in millions of people’s deaths among confirmed cases based on the statistics of the WHO^2^. By July 2023, nearly 700 million confirmed cases, and almost 7 million confirmed deaths were recorded in the world^3,4^. Most patients with the virus experience mild to moderate respiratory illness and heal without needing special treatment. But, some suffer from complications and need medical attention. Older people and those with underlying medical conditions like cardiovascular disease, diabetes, chronic respiratory disease, or cancer are more likely to develop serious illnesses. Anyone can get sick with COVID-19 and become seriously ill or die at any age^5^.

Although the last diagnosis of COVID-19 depends on transcription-polymerase chain reaction (PCR) tests, in states of people with intensive respiratory symptoms the diagnosis protocol depends on medical imaging, which helps doctors to recognize the disease as the sensitivity of PCR is strongly variable^6^. As chest radiography imaging such as computed tomography (CT) imaging and X-ray have been used successfully for the diagnosis of pneumonia, they have a high sensitivity for the diagnosis of COVID-19^2^. The suspected case undergoes an X-Ray session and if more details are required, a computed tomography scan (CT-scan) session is taken. Therefore, X-ray^7^ and CT scan images^8^ are being used as diagnostic methods for COVID-19 and to detect the effects^9^ of the virus^6,10^. The availability and accessibility of X-ray imaging in many imaging centers and clinics is more present even in rural regions as it is standard equipment in healthcare systems. Particularly, chest X-ray is more readily available than CT, because CT scanners require high equipment and maintenance costs. CT is not very suitable for COVID-19 screening as well because of its cost, imaging time, and radiation exposure whereas X-ray is more cost and time effective in dealing with such a common virus^11^.

The abnormalities can only be explained by expert radiologists. With the huge number of suspected cases and the limited number of available radiologists, automatic methodologies for the recognition of these precise abnormalities can aid in early diagnosis with high accuracy. The studies in Artificial Intelligence (AI) and machine learning, especially Deep Learning (DL), achieved high performance in the diagnosis of medical images. Therefore, DL techniques are robust tools for such issues.

Deep learning (DL) has been successfully used to predict COVID-19 from Chest images. Unlike the traditional machine learning techniques DL can be used to predict disease from raw images without feature extraction required. The role of deep learning is to learn the features using a trained model with a huge amount of data to improve the classification's accuracy which reduces the burden on physicians and decreases the effect of doctors’ shortages of the struggle against the disease. Convolutional neural network (CNN) is the type of DL model intended for image analysis tasks and has already been utilized in many medical problems such as segmentation and classification^12^.

Many high-performing pre-trained CNN structures have been provided in the literature to be utilized in similar problems. These models were trained using ImageNet data which contains 1,000,000 images and 1000 classes to overcome the limitation of data and to reduce the training time^13^. These models can be used for image recognition based on transfer learning after fine-tuning these networks to the new problems. The learned weights of these pre-trained models are provided and used directly in the new problems^14^. The purpose of utilizing pre-trained models is to take advantage of learned features on a larger dataset, therefore the new model can converge faster and perform better with a smaller dataset. This gives us the advantage of DL independence of feature engineering over traditional methods without giving up the time, computational resources and cost effeciencies. Examples of these pre-trained CNN models are visual geometry group VGG (16, 19)^15^, EfficientNet (B0 to B7)^16^, MobileNet^17^, and residual neural network (ResNet)^18^, etc.

The contributions of this research can be summarized as follows:

- A framework has been developed to diagnose COVID-19 using chest X-ray images for both full and segmented images.
- A multiplication between each original image and the associated lung mask from the ground truth dataset provided by the database has been applied to get the segmented lung.
- Different image enhancement techniques have been applied to both full and segmented X-ray images to reach the best possible classification performance.
- CNN pre-trained models based on transfer learning have been used to classify both full and segmented chest X-ray images with all enhancement versions and achieved promising results.
- Since the purpose of utilizing pre-trained models is to take advantage of learned features on a larger dataset, therefore the smallest possible datasets that can achieve the best possible performance have been used for faster convergence.

Recently, many works have been developed to detect and diagnose COVID-19 and other lung diseases based on different medical image modalities using different machine learning techniques especially deep learning and transfer learning techniques. The purpose of all these works is to improve the performances of the methodologies used in the detection and classification of COVID-19 and other lung diseases. The focus in the research will be in X-ray images as the adopted medical image modality in this research.

The rest of the paper is organized as follows. Related COVID-19 articles using deep learning are reviewed in the “Related work” section. Then, the proposed framework for COVID-19 classification is described in "The proposed framework" section. Next, the results of X-ray images obtained with the proposed framework are presented in "Experimental results" section. The discussion and comparison with literature are provided in "Discussion" section. Finally, the main “Conclusions and future work” are outlined.

## Related work

Recently, many works have been developed to detect and diagnose COVID-19 and other lung diseases based on different medical image modalities using different machine learning techniques especially deep learning and transfer learning. The purpose of all these works is to improve the performances of the methodologies used in the detection and classification of COVID-19 and other lung diseases. Where the proposed research will use X-ray images as a medical image modality, the focus in this section will be on the previous work based on X-rays.

Nishio, et al.^19^ presented a system based on VGG16 to classify images of chest X-rays as healthy, COVID-19 pneumonia, and non-COVID-19 pneumonia. They applied the proposed system to 1248 X-ray images collected from 2 different public datasets. The collected X-ray images contain 500 healthy samples, 215 images for COVID-19 pneumonia patients and 533 images for non-COVID-19 pneumonia patients. The achieved accuracy was 83.6%, while the sensitivity was 90.9%.

Minaee et al.^20^ applied deep learning to recognize COVID-19 cases using chest X-rays images. Transfer learning was used to train 4 CNN models which are DenseNet-121, SqueezeNet, ResNet50, and ResNet18 to binary classify images as COVID-19 or not. The training was applied to 84 (420 after augmentation) COVID-19 images and 2000 non-Covid images, while the test was applied to 100 COVID-19 images and 3000 non-COVID images. The best achieved sensitivity of these models was 98%, while the specificity was 92.9% for the SqueezeNet model.

Sahin^21^ proposed a CNN model for binary classification of COVID-19 cases as COVID and Normal using chest X-ray images. Also, two pre-trained models which are ResNet50 and MobileNetv2 are applied to the used dataset of 13,824 X-ray images. The proposed CNN model achieved an accuracy of 96.71% and F1-score of 97%. MobileNetv2 achieved an accuracy of 95.73% and F1-score of 96%, while ResNet50 achieved an accuracy of 91.54% and F1-score of 91%.

Wang et al.^22^ developed an open-source CNN called COVID-Net to detect COVID-19 cases using chest X-ray images. The proposed net can predict the case as one of three classes which are COVID-19 viral infection, non-COVID-19 infection, and normal. Also, an open access benchmark dataset COVIDx was introduced, it contains 13,975 X-ray images collected from 13,870 patients. The COVIDx dataset was generated using five different publicaly available datasets. The accuracy of COVID-Net reached 93.3%.

Panwar et al.^23^ developed a deep learning model called nCOVnet for detecting COVID-19 based on X-rays. A dataset of 284 X-ray images was used of which 142 images are normal cases and 142 images are COVID-19 cases. The model achieved an accuracy of 88.1%.

Nigam et al.^24^ used transfer learning to utilize 5 pre-trained models which are DenseNet121, NASNet, Xception, VGG16, and EfficientNet to classify Coronavirus suspected cases as normal, COVID-19 positive cases, and other classes. The used dataset contains 16,634 X-ray images, 6000 normal images, 5634 COVID images, and 5000 imges for others. The achieved accuracies were 79.01%, 85.03%, 88.03%, 89.96%, and 93.48% for VGG16, NASNet, Xception, DenseNet121, and EfficientNet respectively.

Chow et al.^25^ used transfer learning to utilize 18 CNN models including VGG-19, VGG-16, ShufeNet, SqueezeNet. etc. to classify the cases as normal or COVID-19. The used dataset contains 700 X-ray images (350 normal cases and 350 COVID-19 cases) from both public and private institutes. The highest 4 models are VGG-19, VGG-16, ResNet-101, and SqueezeNet with accuracy ranging from 90.7 to 94.3% and F1-score from 90.8 to 94.3%. The VGG-16 is the highest with an accuracy of 94.3% and F1-score of 94.3%. The majority voting of the 18 models and the highest 4 models achieved an accuracy of 93.0% and 94.0%, respectively.

## The proposed framework

In this section, the proposed framework has been explained. First, the used chest X-ray dataset has been described. Then, the developed framework, which includes “pre-processing” phase and the “Classification using CNN models based on transfer learning” phase, has been illustrated. Two different approaches have been used to train pre-trained CNN models using transfer learning. The first approach uses whole chest X-ray images, while the other approach uses lung-segmented images.

### Used datasets

**Figure 1:**
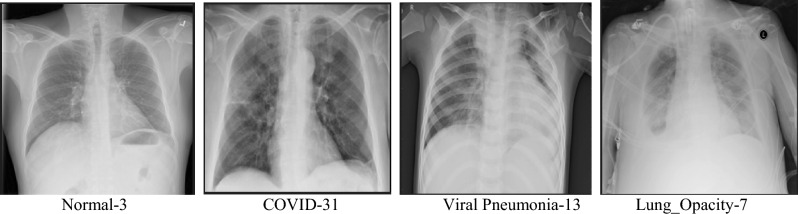
Samples from COVID-19 radiography chest database representing different classes.

In this research, the data obtained from the “COVID-19 Radiography Database” has been used to apply the proposed framework. The database contains thousands of publicly available benchmark X-ray images and corresponding lung masks. The X-ray images are provided in Portable Network Graphics (PNG) format with a resolution of 299 × 299 pixels. The database includes 10,192 Normal cases, 3616 positive COVID-19 cases, 1345 Viral Pneumonia cases, and 6012 Lung Opacity images as shown in Table1. This database was developed by a team from Qatar University, Dhaka University, Bangladesh with cooperators from Malaysia and Pakistan and cooperators of medical doctors^26^. Figure 1 illustrates samples from different classes in the COVID-19 Radiography Database.

**Table 1:** COVID-19 radiography database distribution.

Classes	Normal	Positive COVID-19	Viral pneumonia	Lung opacity
Number of cases	10,192	3616	1345	6012

### Preprocessing

The purpose of the pre-processing phase is to prepare the X-ray images for classification using CNN pre-trained models. In this phase, different pre-processing steps are applied to improve the performance of the classification. The pre-processing steps can be summarized as follows:

#### Image enhancement

Enhancing images is a significant step for the correct classification. It increases image contrast in order to improve classification performance. Different techniques can be applied to enhance the images. In this research, some of these techniques have been applied to the original X-ray images before introducing them to the classification models, they are as follows:

1. Histogram Equalization (HE): The purpose of histogram equalization (HE) is to spread the gray levels inside the image. It modifies the brightness and contrast of the images to improve the image quality^27^. The original X-ray images’ intensity has been enhanced using histogram equalization (HE).
2. Contrast Limited Adaptive Histogram Equalization (CLAHE): It originated from Global Histogram Equalization (GHE), it is based on dividing the image into non-overlapping blocks, and after that, the histogram of each block is gotten using a pre-specified value^28^. In this research, CLAHE has been used to enhance the contrast of original X-ray images.
3. Image Complement: The complement or inverse of X-ray images transforms the dark positions to lighter and the light positions to darker. As this is a standard process, which is similar to that used by radiologists, it may aid a deep learning model for improving classification performance. The complement of the binary image can be obtained by changing the zeros to ones and ones to zeros. Whereas for a grayscale image, each pixel is subtracted from 255.

**Figure 2:**
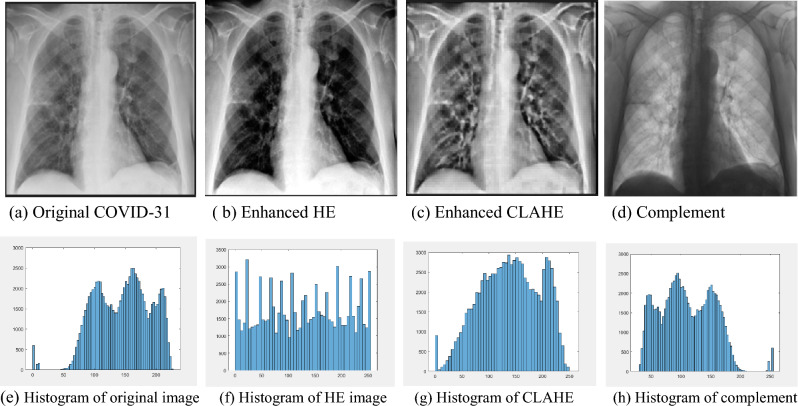
An X-ray image and its enhanced versions after applying HE, CLAHE and complement to the original image and the corresponding histogram plots.

Figure 2 shows an original X-ray image and its enhanced versions after applying HE, CLAHE and image complement on the original image with the corresponding histogram plots for each version.

#### Segmentation

**Figure 3:**
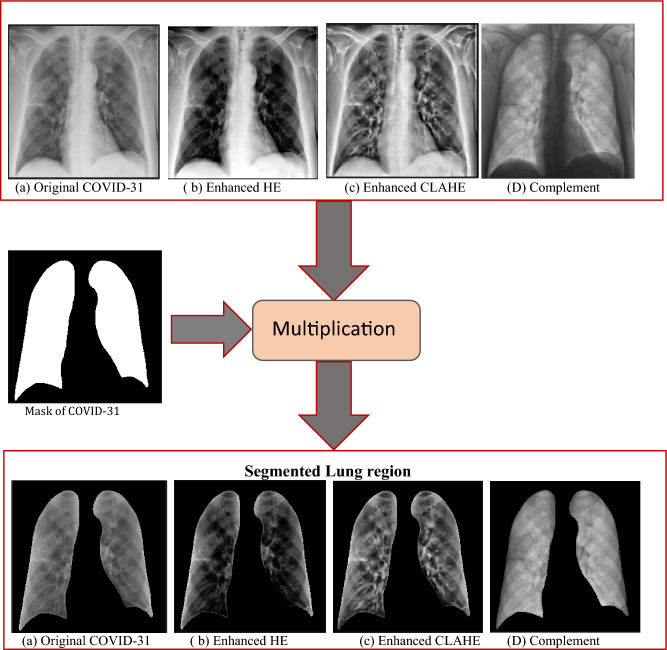
X-ray original image and its enhanced versions and the segmented lung region of each version.

In the segmentation step, the regions of interest (ROI), which are the lungs region in our case, are cropped from the associated image. In this research, the ground truth lungs’ masks which are provided by the database have been used. A modified U-Net model was applied by the authors of the database on the X-ray images to get the lung masks associated with the full X-ray images. In this research, multiplication between each original image and the associated lung mask has been applied to get the segmented lungs. The same process of multiplication between different enhanced image versions and the associated masks has been applied to get different versions of segmented datasets with different enhancements. All these versions are introduced to CNN models as segmented versions of data. Figure 3 shows the segmented images of the original image and of the different enhanced images for one of the COVID samples.

#### Resizing images phase

Resizing the images is an essential process to satisfy the requirement of CNN of equally sized input images. In this research, the process of resizing X-ray images has been done to fit all X-ray images to the input size of the used pre-trained CNN models which are VGG19 and EfficientNetB0. Therefore, all images’ versions either full or segmented versions were resized to fit the CNNs input image size which is 224 × 224 pixels. To expedite the training process, it was found that the size of 112 × 112 pixels expedited the training without affecting the performance metrics.

### Classification using pre-trained convolution neural network model

In this research, different versions of either full or segmented chest X-ray images have been introduced to CNN models to train the classifiers. Different experiments have been carried out on the original and segmented lung X-ray images both with their different enhanced versions. The classification has been done using VGG19^14^ and EfficientNetB0^16^ pre-trained CNN models. After the calculation of different performance metrics, the best model has been selected as the adopted model. The next subsections give a brief description of the used pre-trained models.

#### VGG19 model

VGG19 is a variant of the VGG CNN model which was created by Visual Geometry Group (VGG) at Oxford University. VGG19 was one of the winners of the Image Net Large Scale Visual Recognition Challenge (ILSVRC) in 2014. The size of the input image to VGG19 is (224 × 224). VGG19 contains 16 convolution layers, 5 max-pooling layers and 3 fully connected layers. The convolution layers are with (3 × 3) filters' size, stride of 1 pixel and padding of 1 pixel. The max-pooling layers are with a size of 2 × 2 and a stride of 2. The rectification (ReLU) activation function is utilized for all hidden layers. Then, the first 2 fully connected layers with 4096 channels each are uitilized followed by the last layer of 1000 channels to represent the different 1000 classes of the ImageNet with soft-max activation function^15^.

#### EfficientNetB0 model

**Figure 4:**
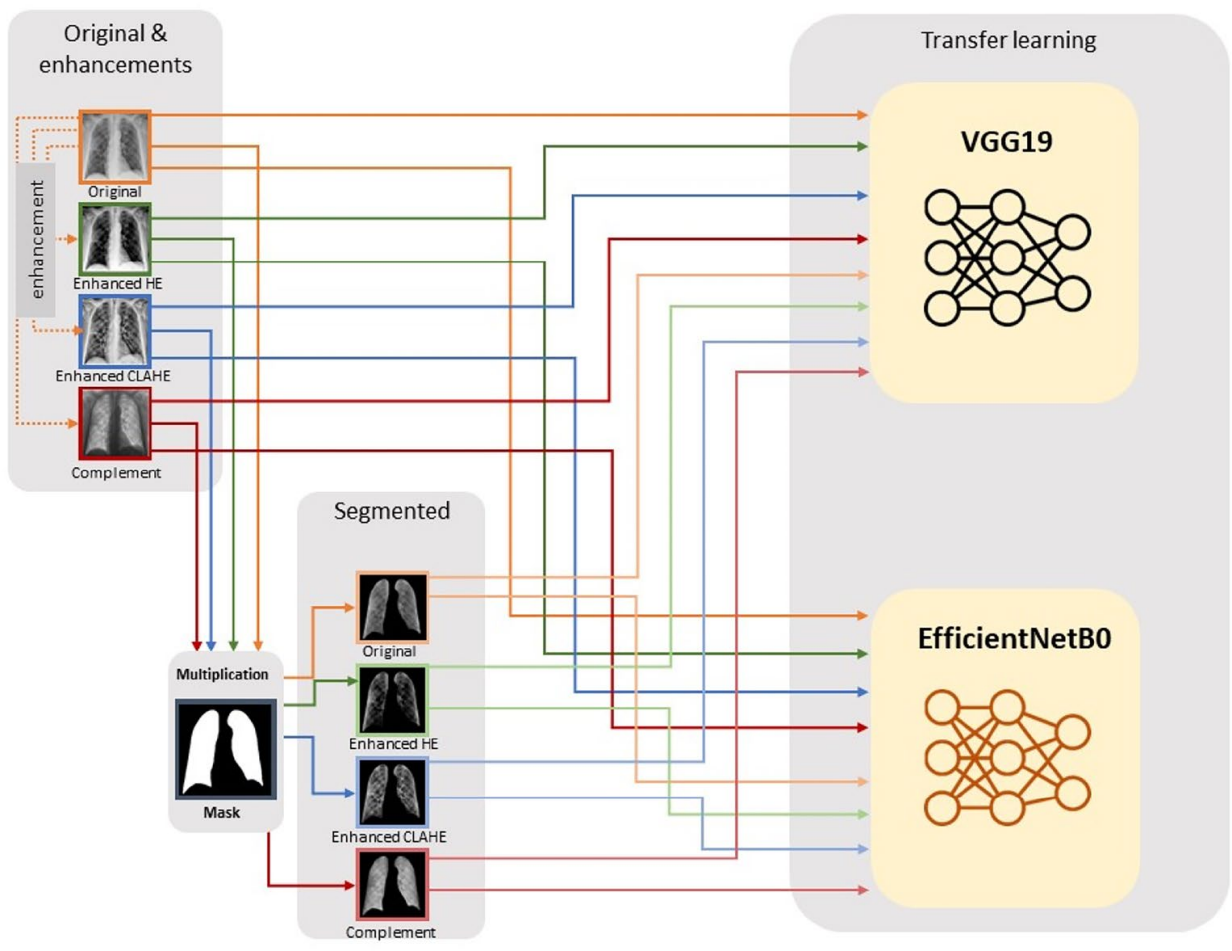
The framework of the used methodology for Chest X-ray images classification.

Google research group designed a family of models, called EfficientNets using a scaling method and achieved better efficiency and accuracy than previous ConvNets. EfficientNet is based on scaling CNNs and reaching better performance by balancing network width, depth, and resolution. Therefore, the focus is to present a scaling method to uniformly scale the 3 dimensions with a simple highly effective compound coefficient. Thus, it can be considered as an optimization problem to find the best coefficients for depth, width, and resolution that maximizes the accuracy of the network given the constraints of the available resources. The primary building block of the EfficientNet models is MBConv. The network's dimension equation was used to get the family of neural networks EfficientNet-B0 to B7^16^. In this research, EfficientNetB0 was used for the classification of the chest X-ray images. Figure 4 sums up the framework of the adopted methodology in this research.

### Ethical approval

This article does not contain any studies with human participants or animals performed by the author.

## Experimental results

This section presents the experimental results of the proposed framework to evaluate its performance and study the effect of different enhancements applied to the X-ray images on the performance of the CNN classification model.

### Experimental setup

Keras Python deep learning library on top of TensorFlow was utilized for implementing CNN models on a machine with the following specification; an Intel^®^ Core™ i7 CPU@ 3.6 GHz with 32 GB RAM and a Titan × Pascal Graphics Processing Unit (GPU). Extensive experiments were carried out to obtain the best settings of the CNN models that achieve the best possible results. It is worth noting that the pre-processing for enhancing the images has been carried out using MATLAB^®^18 software.

**Figure 5:**
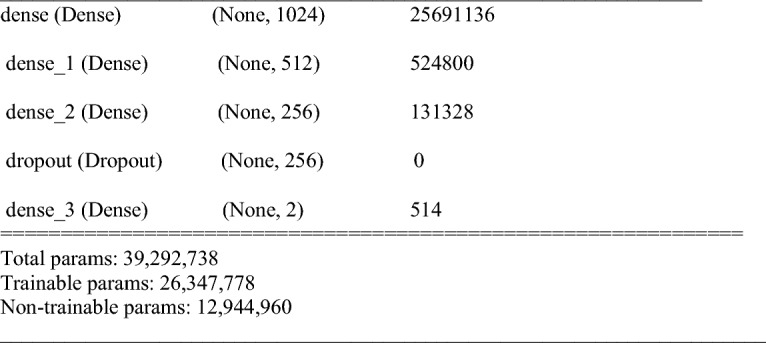
Fine-tuned top layers based on VGG19 pre-training model.

**Figure 6:**
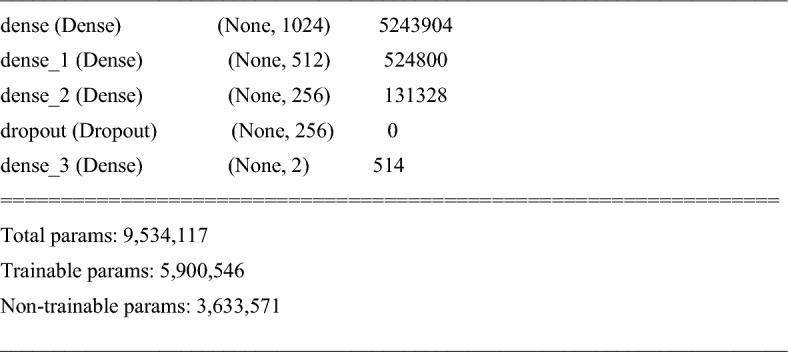
Fine-tuned top layers based on EfficientNetB0 pre-training model.

Both the full and segmented datasets with their enhanced versions have been used to train VGG19 and EfficientNetB0 CNN pre-trained models. The training was carried on with Adam optimizer, learning rate of 0.001, batch size of 32, and the number of epochs (10–30) epochs, SoftMax classifier. The fine-tuned pre-trained models were used for feature extraction; therefore, the weights of the pre-trained models were frozen, and they were not updated during the training to maintain ImageNet’s initial weights. The top layers were fine-tuned to adjust the network according to the used chest X-ray data and to the current problem output which is (2/4) rather than 1000 in the ImageNet data. To avoid overfitting, a dropout of 0.3 was applied in the fully connected layers. Figures 5 and 6 illustrate the fine-tuned top layers based on VGG19 and EfficientNetB0 respectively for binary classification.

### Performance evaluation

A benchmark dataset was employed to validate the performance of the proposed framework. For binary classification, a set of 1600 X-ray images (800 of each class) have been used to train CNN models using transfer learning. The dataset has been divided into three subsets training, validation, and test sets. The training set is used for learning the model and adjusting the parameters. The validation set is to test the model during the training phase and fine-tune the parameters. The test set is to evaluate the trained model. The division was done as 400 samples (25%) of the used X-ray images were selected randomly for testing (200 images for each class), and the remaining 75% samples were split again into training and validation splits (80–20%). For 4-classes classification, a set of 3200 X-ray images (800 of each class) have been used. Before training CNN models, different pre-processing steps were implemented to enhance the images of both full and segmented lungs chest X-ray images to investigate the classification performance of the CNN models using the different versions.

The following metrics were used for the evaluation of the different CNN models trained using various dataset versions:

1 $${\text{Sensitivity}}/{\text{Recall}}(\mathrm{\%})= \frac{{\text{TP}}}{{\text{TP}}+{\text{FN}}}$$

2 $$\mathrm{Precision }(\mathrm{\%})=\frac{{\text{Tp}}}{{\text{TP}}+{\text{FP}}}$$

3 $$\mathrm{Specificity }\left(\mathrm{\%}\right)=\frac{{\text{TN}}}{{\text{TN}}+{\text{FP}}}$$

4 $$\mathrm{Accuracy }(\mathrm{\%})= \frac{{\text{TP}}+{\text{TN}}}{{\text{TP}}+{\text{FP}}+{\text{TN}}+{\text{FN}}}$$

5 $${\text{F}}1\mathrm{ score}=\frac{2\mathrm{ TP}}{2{\text{TP}}+{\text{FP}}+{\text{FN}}}$$

where TP is a true-positive value, FP is a false-positive value, TN is a true-negative value and FN is a false-negative value.

## Results

Tables 2 and 3 show the results of training VGG19 using the original and different enhanced versions of full X-ray images and of segmented versions respectively. As it is shown in Table 2, applying different image enhancement techniques has improved the performance of the classification model. The accuracy of classification for the model trained using the original images’ version was 0.913, however, it has been improved for the enhanced versions to reach 0.94, 0.95, and 0.9475 for histeq, CLAHE, and complement respectively. The detailed results including TP, TN, FP, FN, sensitivity, specificity, precision, F1 score, test accuracy, and AUC of the proposed full X-ray images using VGG19 are shown in Table 2. The performance of the model trained using the CLAHE version is the best.

**Table 2:** The results of applying VGG19 to original and different enhanced versions of the used dataset.

Dataset	Tn	Fp	Tp	Fn	Sensitivity	Specificity	Precision	F1 score	Test acc	AUC
Original	170	30	195	5	0.975	0.85	0.8667	0.9176	0.913	0.9125
Histeq	192	8	184	16	0.92	0.96	0.9583	0.9388	0.94	0.94
CLAHE	188	12	192	8	0.96	0.94	0.9412	0.9505	0.95	0.95
Complement	187	13	192	8	0.96	0.935	0.93659	0.9482	0.9475	0.9475

**Table 3:** The results of applying VGG19 to original and different enhanced versions of the used dataset after segmentation.

Dataset	Tn	Fp	Tp	Fn	Sensitivity	Specificity	Precision	F1 score	Test acc	AUC
Segmented original	157	43	198	2	0.99	0.785	0.8216	0.898	0.887	0.8875
Segmented Histeq	173	27	191	9	0.955	0.865	0.876	0.9139	0.91	0.91
Segmented CLAHE	173	27	189	11	0.945	0.865	0.875	0.9086	0.905	0.9049
Segmented complement	169	31	194	6	0.97	0.845	0.862	0.9129	0.908	0.9075

Regarding the segmented versions, as it is shown in Table 3 the accuracy of classification of the model trained using the original segmented dataset version was 0.887. However, the accuracy has been improved for the enhanced segmented dataset versions to reach 0.91 using Histeq techniques, 0.9049 for CLAHE and 0.9075 for the complement version. It is clear that the accuracies using different enhanced versions are close to each other, and they are better than that of the original segmented version. The detailed results using the different metrics are shown in Table 3.

Table 4 shows the results of training EfficientNetB0 using the original and different enhanced versions of full X-ray images. The accuracy of classification using the original full images’ version was 0.915, it reached 0.94, 0.938, and 0.94 for histeq, CLAHE, and complement versions respectively. The accuracies for the models trained using different enhanced versions are better than that of the original version. The detailed results using the different metrics are shown in Table 4.

**Table 4:** The results of applying EfficientNetB0 to original and different enhanced versions of the used dataset.

Dataset	Tn	Fp	Tp	Fn	Sensitivity	Specificity	Precision	F1 score	Test acc	AUC
Original	173	27	193	7	0.965	0.865	0.877	0.91905	0.915	0.915
Histeq	192	8	184	16	0.92	0.96	0.9583	0.9387	0.94	0.94
CLAHE	194	6	181	19	90.5	0.97	0.9679	0.9354	0.938	0.9375
Complement	193	7	183	17	0.915	0.965	0.963	0.9385	0.94	0.94

Regarding the segmented versions, as shown in Table 5 the accuracy of the classification for the EfficientNetB0 model trained using the original segmented lung dataset version was 0.885. However, the accuracy has been improved to 0.905, 0.905, and 0.9075 for Histeq, CLAHE and complement versions respectively. As with VGG19, the accuracies of training EfficientNetB0 using different enhanced segmented versions are close to each other, but they are all better than that of the original segmented version. The detailed results using the different metrics are shown in Table 5.

**Table 5:** The results of applying EfficientNetB0 to original and different enhanced versions of the used dataset after segmentation.

Dataset	Tn	Fp	Tp	Fn	Sensitivity	Specificity	Precision	F1 score	Test acc	AUC
Segmented original	160	40	194	6	0.97	0.8	0.829	0.894	0.885	0.885
Segmented Histeq	181	19	181	19	0.905	0.905	0.905	0.905	0.905	0.905
Segmented CLAHE	183	17	179	21	0.895	0.915	0.9133	0.904	0.905	0.905
Segmented complement	174	26	189	11	0.945	0.87	0.879	0.9108	0.9075	0.9075

It is clear that the performance of all enhanced versions is better than that of their associated original version using either VGG19 or EfficientNetB0 models and for both full and segmented versions. By comparing the results of the full X-ray images and segmented images using the same CNN model, reductions in the performance of the CNN models trained using the segmented datasets rather than those trained using full-image datasets were observed. The models built using full images achieved better performance in general. The reason for that might be that the full images may have more details outside the lung region in the surroundings region that contribute to the classification and help to improve the performance.

**Figure 7:**
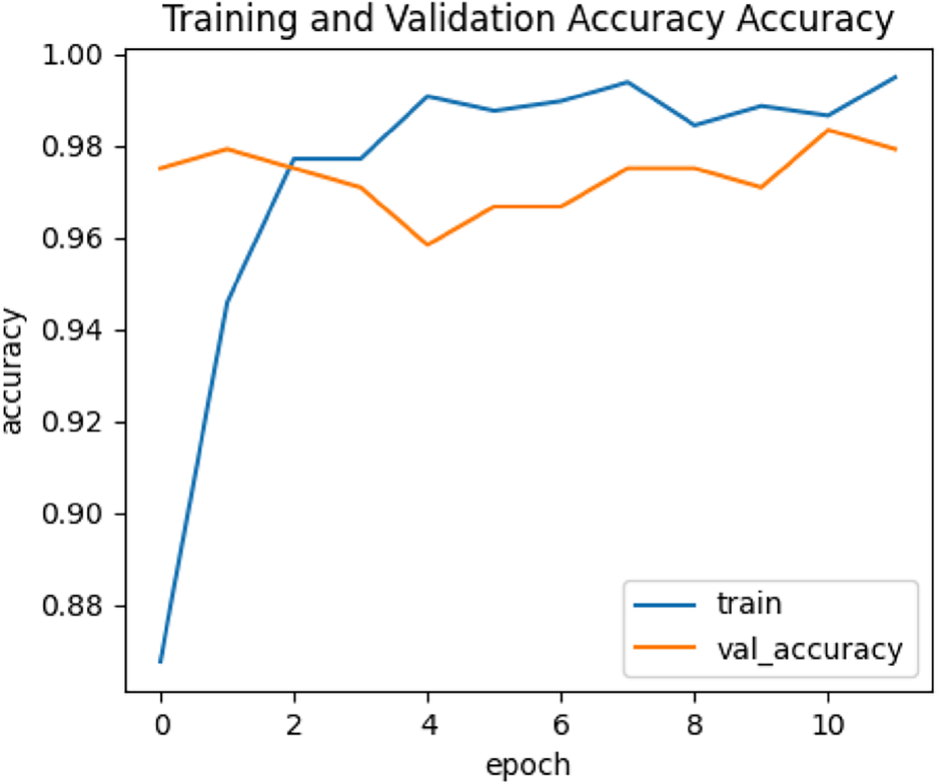
Training and validation accuracy of the best model.

**Figure 8:**
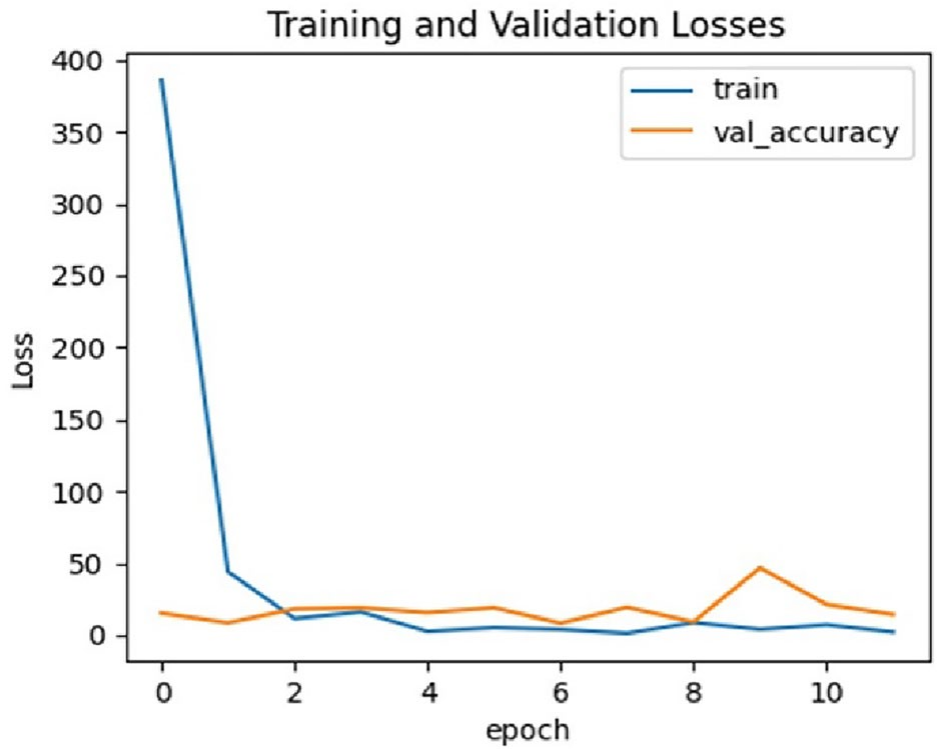
Training and validation losses of the best model.

**Figure 9:**
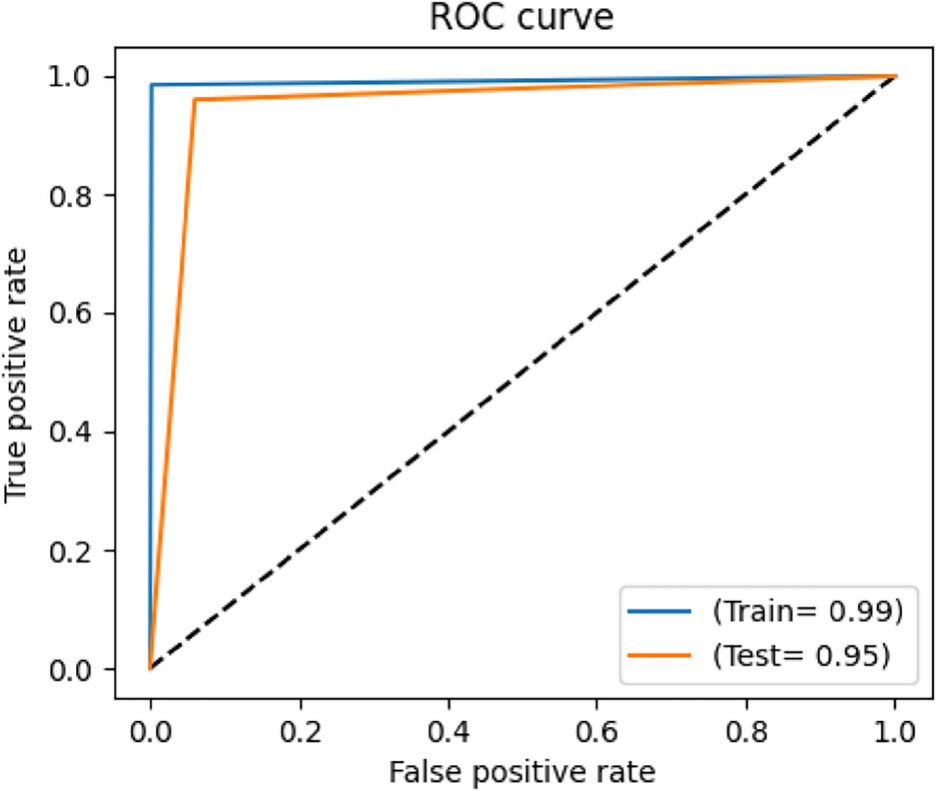
Training and test ROC curve of the best achieved model.

**Figure10:**
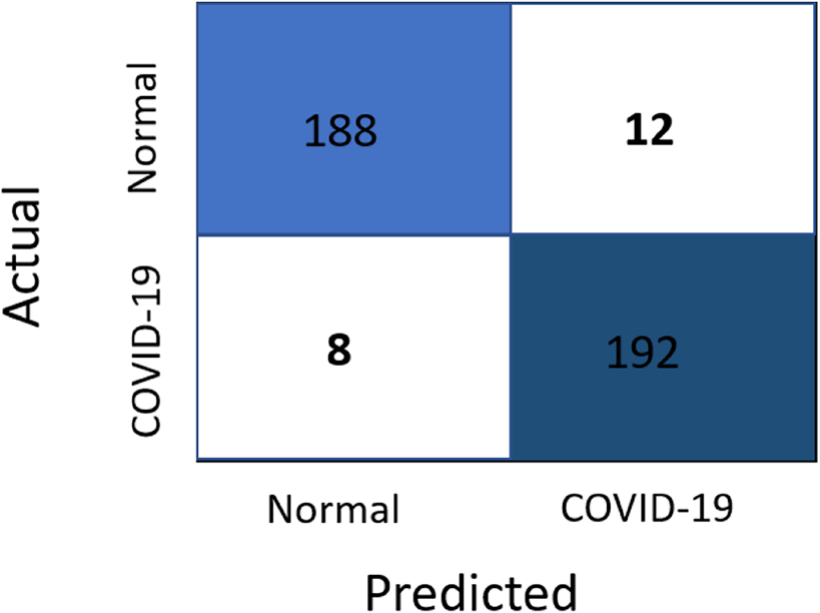
Confusion matrix of the best model.

Regarding the comparison of the used 2 CNNs models, the results of the two models are close to each other, however, the results of VGG19 are a bit better than those of EfficientNetB0. The best achieved performance is of the VGG19 model trained using the CLAHE version of full X-ray images for binary classification of chest X-ray images into COVID-19 or normal. It achieved a sensitivity of 0.96, specificity of 0.94, precision of 0.9412, F1 score of 0.9505 and accuracy of 0.95. Figures 7, 8, 9 and 10 show the training and validation accuracies, the training and validation losses, the training and test ROC curve and the confusion matrix of that best achieved model respectively.

## Discussion

Distinguishing COVID-19 from normal and other classes is one of the important issues since the pandemic in 2019. The contribution of this research is to develop a framework to classify Coronavirus suspected cases as normal or COVID-19 positive cases. Different pre-processing steps have been applied to improve the performance of the classification. After that, multiplication between the original images and the associated lung masks has been applied to get the segmented lungs. The same process of multiplication has been applied between different enhanced image versions and the associated masks to get different enhanced versions of segmented datasets. All these versions are introduced to CNN models which are VGG19 and EfficientNetB0. Therefore, two different approaches have been used to train pre-trained CNN models using transfer learning. The first approach uses full chest X-ray images, while the other approach uses lung segmented images.

From the results of conducted experiments, it has been observed that the proposed framework has achieved a good performance using either full or segmented images, however the performance using full images is better than that using segmented. Moreover, it has been observed that the performance of the classification models has been improved after applying enhancement techniques.

To evaluate the proposed framework with respect to the state-of-the-art works in COVID-19, it has been compared with the related works reviewed in this research as described in Table 6. It is worth mentioning that, the comparison is not an easy task in COVID research as the pandemic broke out in the world suddenly, all Covid research used different sources of data either local, public, or combined from different databases. Some of the public datasets are collected from different other databases. Even the research that used the same public datasets used different number of samples. Some research performed binary class classification, where others performed multi-class classification. Thus, the proposed work has been compared with others that used the same modality which is X-ray with mentioning the number of used samples and the task.

**Table 6:** Comparison of proposed method with the relevant researches.

Ref	Techniques	Modalities	Task	Accuracy
^19^	CNN (VGG16)	1248 chest X-ray	3-classes classification: (Healthy/COVID-19pneumonia/non-COVID-19 pneumonia)	83.6% accuracy, 90.9% sensitivity
^20^	SqueezeNet	5184 Chest X-rays	Binary classification of images as COVID-19 or not	98% sensitivity, 92.9 specificity
^21^	CNN model	13,824 X-ray images	Binary classification of images as COVID-19 or normal	96.71% accuracy, 97% F1-score
^22^	CNN called COVID-Ne	13,975X-ray	Prediction of cases as (normal/pneumonia/COVID-19)	93.3% accuracy
^23^	CNN called nCOVnet	284 X-ray images	(Normal/COVID-19)	88.1% accuracy
^24^	EfficientNet	16,634 X-ray images	Classify cases as: (Normal/COVID-19/other)	93.48% accuracy
^25^	VGG-16	700 X-ray images	binary classification (Normal/COVID-19)	94.3% accuracy, 94.3% F1-score
Proposed model	VGG19	1600 X-ray images	Binary classification (Normal/COVID-19)	95% accuracy, 0.9505% F1-score, 0.96% sensitivity, 0.94% specificity

By comparing the results of the proposed framework with recent literature, it was found that the proposed framework outperforms most of the state-of-the-art works. However,^21^ slightly outperforms the proposed framework. Where the accuracy and F1-score of^21^ are 96.71% and 97% respectively, the corresponding values of the proposed framework are 95% and 0.9505 respectively. Taking in consideration that in^21^ un-balanced data has been used; the number of COVID images used is 3626 where the number of normal images is 10,198 as mentioned in the manuscript.

For more validation, different classes of the datasets have been used for training the CNN models. To check the capability of the proposed framework for 4-classes classification. The dataset versions that achieved the highest binary classification have been utilized for multi-classes classification. Since the performance of all models that trained using enhanced full image versions is close to each other, therefore, these versions have been utilized for 4-classes classification. A set of 3200 X-ray images (800 of each class) have been used to train CNN models. The newly added classes are Viral Pneumonia and Lung Opacity in addition to COVID-19 and normal classes.

It was found that the best-achieved accuracy of 4-classes classification using the full image versions reached 0.935 for histeq version by EfficientNetB0. While it reached 0.93375 for both CLAHE, and complement versions using VGG19. Table 7 shows the results of the best-achieved models for 4-classes classification.

**Table 7:** The results of best achieved models for 4-classes classification.

Dataset	Model	F1 score	Test acc
Histeq	EfficientNetB0	0.93495	0.935
CLAHE	VGG19	0.9339	0.93375
Complement	VGG19	0.9337865	0.93375

## Conclusion and future work

In this research, a framework has been developed for automatically classifying chest X-ray images as COVID-19 positive cases or normal cases. Different techniques such as histeq, CLAHE, and complement have been applied to enhance the original X-ray images and therefore, both the original and enhanced versions have been introduced to the selected CNN pre-trained models. Two pre-trained CNN models which are VGG19 and EfficientNetB0 have been used to train different versions with the last dense layer set to (2/4) according to the number of classification classes.

Two approaches have been utilized to train pre-trained CNN models which are using whole chest X-ray images and using lung segmented images with their enhanced versions. The best binary classification accuracy reached 95% for the model trained using CLAHE full images version utilizing VGG19. The best achieved accuracy for a model trained using a segmented dataset is 91% for the model trained using Histeq version utilizing VGG19. By testing the framework for 4-classes classification, it achieved promising results which reached 0.935 accuracy.

It is obvious from the results that, the proposed framework can be employed in the future to support physicians and decrease the effect of doctors’ shortages in the struggle against the disease. However, extra validations are required before applying any system, as more accuracy and more careful experiments are needed when things are related to human life. In the future, the authors are willing to try the proposed model on local data.

## Data Availability

The used data has been obtained from an available online database and it has been referenced in the manuscript. The link to the database used in the study: https://www.kaggle.com/tawsifurrahman/covid19-radiography-database
